# 45,X/47,XXX Mosaicism and Short Stature

**DOI:** 10.1155/2015/263253

**Published:** 2015-06-07

**Authors:** Erica Everest, Laurie A. Tsilianidis, Anzar Haider, Douglas G. Rogers, Nouhad Raissouni, Bahareh Schweiger

**Affiliations:** ^1^Cleveland Clinic, 9500 Euclid Avenue, Cleveland, OH 44195, USA; ^2^Case Western Reserve University School of Medicine, 10900 Euclid Avenue, Cleveland, OH 44106, USA

## Abstract

We describe the case of a ten-year-old girl with short stature and 45,X/47,XXX genotype. She also suffered from vesicoureteric reflux and kidney dysfunction prior to having surgery on her ureters. Otherwise, she does not have any of the characteristics of Turner nor Triple X syndrome. It has been shown that this mosaic condition as well as other varieties creates a milder phenotype than typical Turner syndrome, which is what we mostly see in our patient. However, this patient is a special case, because she is exceptionally short. Overall, one cannot predict the resultant phenotype in these mosaic conditions. This creates difficulty in counseling parents whose children or fetuses have these karyotypes.

## 1. Introduction

Turner syndrome (TS) is a condition characterized by short stature (nearly all girls are <5 feet tall), sexual underdevelopment, a webbed neck, and cubitus valgus (forearm angled away from the body), resulting from a 45,X cell line [1, 2]. Further features may include edema of the hands and feet, characteristic facies, high palate, and short fourth metacarpals [3, 4]. Additionally, renal abnormalities, such as horseshoe kidney, can cause serious health problems [5]. Most concerning are the cardiac abnormalities, such as dilated aortic root, which occur in half of the patients [6]. Women with TS are unlikely to conceive spontaneously, but if they do they are at a high risk of losing the pregnancy or having a baby with congenital anomalies or sex chromosome abnormalities [7]. TS occurs in 1 in 2000 female births [8].

Triple X syndrome is the most common female chromosomal abnormality, occurring in approximately 1 in 1,000 female births, and a review by Tartaglia et al. reveals the subsequent features of the condition [9]. Its most common characteristics are tall stature (>75th percentile), epicanthal folds, clinodactyly, and hypotonia. Possible additional problems can be seizures, renal and genitourinary abnormalities, and premature ovarian failure. The onset of puberty, sexual development, and fertility are usually normal. Also, more common than in the general population are delays and psychological issues, motor and speech delays, learning disabilities, attention deficits, and mood disorders. There is considerable variation in the phenotype with this disorder. This is reflected by the fact that only 10% of cases are diagnosed clinically.

Mosaic forms of TS tend to have improved prognoses and milder phenotypes. The improved growth and ovarian function of 45,X/46,XX patients over 45,X patients have been well established, and the rarer karyotype 45,X/47,XXX (about 2% of those with TS) also results in more mildly affected girls [10]. The study from Glasgow, Scotland, evaluated the seven 45,X/47,XXX girls registered in the Scottish Turner Syndrome database. Three of the seven subjects did not require growth hormone to achieve a satisfactory height, in comparison to the 45,X and 45,X/46,Xi(X)(q10) matched subjects, all 21 of whom required growth hormone. Additionally, all the 45,X/47,XXX subjects underwent spontaneous puberty, and all five of those older than twelve had spontaneous menarche with regular menstrual cycles. Only 2 of the 14 girls in the 45,X comparison group had spontaneous puberty, and none achieved menarche without the use of estrogen. This is consistent with previous findings [11, 12]. Also, none of the 45,X/47,XXX girls had cardiac or renal abnormalities, though two had middle ear issues. This is in contrast to the 13 of 21 matched subjects with abnormalities to the heart, renal system, or both and is in contrast to the 15 of 21 girls with middle ear issues. The study also had a geneticist, who was blinded to the genotype of the subjects, evaluate for dysmorphic features. 45,X/47,XXX subjects had the most mild expression. Lastly, none of the 45,X/47,XXX subjects had special education needs, in contrast to four in the comparison group. It appears that the haploinsufficiency from the single X chromosome in 45,X is mitigated by the overtranscription of the X chromosome that results from having a 47,XXX cell line [10].

We report a case of a ten-year-old girl with short stature and a history of renal and urinary tract issues. She is a 45,X/47,XXX mosaic. Overall, she represents a mild phenotype, in the fact that she is experiencing few of the Turner stigmata. However, she has significant short stature, and this has not been previously well reported.

## 2. Case Presentation

A ten-year-three-month-old female presented to the Pediatric Endocrinology Clinic for assessment of short stature. At a height of 4 feet and weight of 51 pounds, she is at the 0.54 and 0.86 percentiles for the CDC 2–20-year stature-for-age and weight-for-age data, respectively. Her parents are of typical height; her mother is 5′5′′ (and had menarche at age 12), and her father is 5′11′′. Her past medical history includes vesicoureteric reflux, urinary tract infections, and decreased kidney function. She was treated with Bactrim from 10 weeks to 3 years for vesicoureteric reflux. She had surgery on her ureters in 2008. She has Tanner stage 1 pubic hair and breasts, normal genitalia, and no axillary hair. She has had body odor and acne for a few years. Neither the review of systems nor the physical examination revealed anything out of the ordinary. She is a fifth grader, who is performing well in school.

Laboratory data showed a normal complete blood count and comprehensive metabolic panel. TSH was 2.260 *μ*U/mL (0.400–5.500 *μ*U/mL) and free T4 was 1.3 ng/dL (0.7–1.8 ng/dL). Transglutaminase antibody (tTG) was 1 unit (normal <20 units). Insulin-like growth factor I was 243 ng/mL (76–478 ng/mL). Bone age was read and interpreted as 8 years 10 months according to the standards of Greulich and Pyle [13], which is considered a normal bone age.

Given the normal thyroid function levels, hypothyroidism is not the cause of her growth failure. Celiac disease is also an unlikely cause of her growth failure, because she did not report abdominal symptoms and her tTG was normal. Normal IGF-1 makes growth hormone (GH) deficiency less likely, because GH stimulates IGF-1 production, but does not necessarily exclude it. Despite the patient not having any of the other stigmata of TS, we ordered a karyotype, because sometimes poor growth is the only presenting symptom.

A chromosome analysis was performed. Thirty metaphase cells were examined from synchronized and unsynchronized PHA-stimulated peripheral blood cultures. Three cells had 45 chromosomes with a single X chromosome (45,X). This chromosome complement is associated with TS. Twenty-seven cells had 47 chromosomes with three X chromosomes (47,XXX). No karyotypically normal cells were identified in the specimen. These two cell lines were presumed to have arisen by a nondisjunctional event at a very early stage of fetal development.

## 3. Discussion

While 45,X/47,XXX girls have milder phenotypes, as discussed previously, the outcome for any individual is unpredictable. In the case of our patient, we saw urinary system malformations and short stature, but no cardiac, middle ear, pubertal, or learning issues. We are seeing the early stages of spontaneous puberty, and she will probably experience spontaneous menarche shortly [10].

Our patient has a 1 : 9 ratio of 45,X : 47,XXX karyotypes in the cells examined from her peripheral blood smear. However, this does not necessarily reflect the distribution of cells throughout the organ systems of her body. Moreover, most patients assessed for Turner syndrome have only been karyotyped from one tissue, so we do not know which lines dominate in which organs [2]. Researchers at USC Medical Center reported the case of a patient with short stature whose buccal smear showed 45,X/46,XX/47,XXX in a 67/123/10 ratio, whose peripheral leukocyte culture showed 45,X/47,XXX in a 1/1 ratio, and whose skin fibroblast culture showed 45,X/47,XXX in a 5/19 ratio [14]. They confirmed a previous assertion that the proportions of chromosomally different cell lines have little value for phenotype prediction, because the chromosome makeup is so varied depending on the sample tissue [15]. However, they presumed that the 47,XXX line was dominant because of the minor Turner syndrome stigmata. Yet, the 45,X line determined her height.

In contrast to the notion that the ratios do not have predictive value, Akbas et al. present the theory that patients with the 45,X/47,XXX karyotype demonstrate the phenotypes in proportion to their degree of mosaicism [16]. They present the case of a patient with a 35%/65% 45,X/47,XXX ratio. She has short stature and a horseshoe kidney, but what they otherwise describe as a mild phenotype. They compared their patient to a case of a woman with a 90%/10% 45,X/47,XXX ratio and a severe phenotype. She had streak gonads, amenorrhea, thyroiditis, short stature, and learning difficulties [17]. Therefore, the cell ratio may be predictive when it comes to the overall assessment of mild phenotype versus severe phenotype. However, height is not affected proportionally, as demonstrated from this case and others reported [2]. Given that 47,XXX girls present with increased height, if 90% of our patient's sampled cells were 47,XXX, we would not expect her to be at the 0.54 percentile for height.

Overall, making predictions regarding what the future has in store for these mosaic girls is nebulous. For prenatal diagnosis, parents of 45,X/47,XXX girls should be counseled for the possibility of full Turner symptoms but with optimism for a better outcome. Intellectual impairment is reduced compared to 45,X Turner syndrome, which is an important concern for parents who may be considering selective termination [2]. Future fertility also cannot be guaranteed but can be successful in most 45,X/47,XXX women [18].

## 4. Conclusion

Phenotypes of 45,X/47,XXX mosaic girls are unpredictable. Cell counts that provide a ratio of 45,X cells to 47,XXX cells should not be considered to have predictive value, because they vary by tissue. Fortunately phenotypes tend to be milder, but the height may be quite short in a way that is out of proportion to the relative mildness of the remainder of the phenotype. Fortunately short stature can be corrected with growth hormone, so families should be aware that this is a possible requirement for their girls. More research will need to be done in this area to assess impact of growth hormone on 45,X/47,XXX mosaic female with short stature as this has not been well studied.
